# Beyond polarization for coexistence with biodiversity: Reply to Bruskotter et al. (2025)

**DOI:** 10.1111/cobi.70173

**Published:** 2025-11-19

**Authors:** Simon Pooley

**Affiliations:** ^1^ School of Social Sciences Birkbeck University of London London UK; ^2^ School of Life Sciences University of KwaZulu‐Natal Pietermaritzburg South Africa

I am pleased that my Diversity article (Pooley, 2024) elicited a response from significant thinkers in the field of human–wildlife interactions (Bruskotter et al., 2025). They found things to agree with in this article but are critical of perceived omissions and my alleged intention to “[operationalize] before adequately understanding what it means to coexist with biodiversity—in principle….” Rather than reply with a series of rebuttals, I considered what their reply says about debates over coexistence as the concept enters mainstream conservation and whether coexistence fits in existing narratives or disrupts them.

I disavow the authors’ opening assertion: “Pooley… laments that efforts to identify ‘new universal rules to explain and govern how to live successfully with wildlife’ may ultimately fail” (Bruskotter et al., 2025). I do not lament any such failure because I do not believe such universal rules exist; neither do I believe such universal rules should exist as they too often provide a means to exert unilateral authority and power.

I refer specifically to general rules intended to describe the nature of coexistence with wildlife, everywhere, for uniform application by governments to foster coexistence in every context. For example, a coexistence rule could dictate that any impact of wildlife on humans or vice versa that exceeds a defined threshold, for example, a particular level of harm to physical or economic well‐being, requires action against the offending animal or person. Lethal control of animals that expose humans to imminent danger is widespread—for example, sharks in Australia and Bruno the bear in Germany (Swan et al., 2017).

If a universal law of coexistence decrees that loss of human or animal life or damages to a defined degree or of a specified type (e.g., seals eating salmon in Scottish rivers [Swan et al., 2017]) are incompatible with coexistence and require management intervention, this can or will in some contexts interfere with existing human–wildlife coexistence systems. For example, in parts of East Timor, for spiritual reasons local communities may not want a crocodile that has killed a person to be killed in retaliation, even though this a widely accepted management response. This is challenging for government authorities to manage, and consultation with communities is recommended before action is taken (Brackhane et al., 2019).

My additional concern is that a coexistence standard offers significant opportunities for outside control, including censure and certification of conservation efforts. Certification schemes, whether donor driven or adopted to meet the recommendations of international organizations such as the International Union for Conservation of Nature (Dudley & Stolton, 2021), influence who receives permits and funding to undertake research or conservation interventions.

In an increasingly difficult funding environment for conservation, this further empowers large, Western nongovernmental organizations and others with economic resources and political influence to direct conservation efforts. These actors, rather than governments of Global South countries or local authorities, may be the agents of top‐down governance. Such actors may disallow locally sanctioned practices, such as sustainable use of wildlife, that offend their values (Artelle et al., 2021; Chua et al., 2020).

Arguing that no universal rules akin to the laws of physics exist for human–wildlife coexistence does not imply that governments or the rule of law should be abolished. However, Bruskotter et al. are correct that I emphasized the importance of process justice. Those actually living with the wildlife should be involved in the development of a vision for how they will coexist with wildlife, should they choose to. Bruskotter et al. characterize this as “bottom‐up,” but this need not exclude top‐down governance, as can be seen in the case of government‐supported Community‐Based Natural Resource Management (CBNRM) in Namibia, for example.

I emphasized what has been most missing from conservationist thinking about human–wildlife interaction management, which is involving other stakeholders, rather than persuading or forcing them to adopt (or tolerate) a conservation‐oriented vision. I did not think it needed to be repeated that governments and their designated authorities would continue to oversee management of human–wildlife interactions, especially where risks are posed to either of the parties.

Bruskotter et al.’s criticism that I am ignoring top‐down governance in favor of a bottom‐up approach polarizes the issue and is symptomatic of deeper conflicts in conservation. Readers wishing to understand the wider context of our exchange on this should read an earlier exchange with some of the same authors about the ethics and effectiveness of collaboration in conservation (Pooley & Redpath, 2018; Vucetich et al., 2018).

As a supporter of democracy in a time in which it appears beleaguered, I agree with Bruskotter et al. that “Representative democracies are routinely a mixture of top‐down and bottom‐up governance.” The challenge is to stay with the trouble and continue trying to find a middle way through, which is more difficult than choosing one or the other pole (characterized as anthropocentrism vs. biocentrism) and claiming this as the moral high ground. Notably, Bruskotter et al. raise specific reservations about bottom‐up governance but cite no reservations about top‐down governance (see Vucetich et al. [2018]). They seem skeptical of both the moral value and effectiveness of bottom‐up approaches.

The main impetus for their response seems to be concern about the question of authority over biodiversity. This is raised in the guise of justice, specifically the concepts of procedural justice (fair and transparent processes for arriving at an outcome); outcome justice (fair outcome, whatever the process); and distributive justice (fair allocation of benefits). Bruskotter et al. argue that my inclusive “vision of coexistence appears overly focused on procedural justice at the expense of outcome justice.” This, they imply, favors humans and fails to adequately consider the consequences for biodiversity.

What I advocated in my piece is: “Adherents willingly coadapt and live so that wildlife can persist within agreed boundaries of what the land and its nonhuman inhabitants require to flourish and within what is acceptable, agreed, and necessary for the human communities to persist and flourish” (Pooley, 2024). That seems to me to include favorable outcomes for humans and nonhumans.

I welcome discussion of justice as a necessary dimension of this conversation, but I am cautious when conservationists introduce the idea of justice into discussions about who should be considered when thinking about policies and interventions. Appeals to justice can be mobilized as arguments from a higher authority, referencing a domain beyond the expertise of most.

In the current conservation climate, advocates of such approaches must avoid perceptions of biocentric bias and distrust of democratic processes. Their appeal to justice is therefore grounded in claims to speak both for the unrepresented (animals) and for future generations who depend on the persistence of biodiversity for their own well‐being (Treves et al., 2019; Vucetich et al., 2018). Bruskotter et al. claim to champion democracy by widening the scope of representation when making decisions affecting biodiversity.

No democratic process is proposed for selecting the judges and advocates for biodiversity, and it is unclear to what extent they should consult and collaborate with others. The theatre for their interventions is a notional court, a legal context ruled by a universally applicable body of law and presided over by a judge who will make top‐down decisions that become binding on everyone (Pooley & Redpath, 2018).

When Bruskotter et al. introduce distributive justice, they advocate “wise” judgments, which require wise judges. How are these judges elected and within which (and whose) legal framework should they work? The experience of environmental campaigners in the period of the great acceleration of human impacts on the natural world is that courts of law and international conventions and treaties are dominated by those who can hire the most high‐powered lawyers. Although environmental conventions proliferate and international courts may make landmark rulings, the record of courts enforcing punitive action on the powerful transnational companies and rich developed world countries that contravene these is poor (Hoffman et al., 2022). Further, decisions by international convention bodies, such as trade bans by the Convention on International Trade in Endangered Species, are not universally recognized as legitimate and are therefore resisted, for example, by South African rhinoceros owners (Challender et al., 2025).

Bruskotter et al. segue from what I was advocating to a conservation‐ and biodiversity‐centered framework underpinned by Western conceptions of universal justice. Certainly, conservation science and affiliated disciplines and Western ideas about rights and justice represent key voices and perspectives for discussions of human–wildlife coexistence. However, they are not the only voices.

Bruskotter et al. (2025) maintain that Western academic scholarship on the “moral standing of nonhuman entities” is in its infancy. Although this may be true, certainly in mainstream conservation science and sustainable development contexts, there are lively and venerable traditions outside Western scholarship with much to say about this—for example, the Māori Kaitiakitanga system or the Andean Pachama concept (Hill et al., 2023; Landim et al., 2024). These cannot be dismissed as utopian musings that fail to understand that coexistence is not only about blue‐sky happiness but also involves tough decisions on tolerating harm.

Environmental humanities scholars have been grappling with these issues, including incorporating non‐Western perspectives, for decades (Bird Rose et al., 2012). More importantly, Indigenous thinkers are entering the conservation mainstream (e.g., Nemquino & Anderson, 2024), and the Intergovernmental Science‐Policy Platform on Biodiversity and Ecosystem Services (IPBES, 2022) is working to integrate these into mainstream conservation policy making. At New York University Law, the More‐Than‐Human‐Life (Moth) Program integrates Indigenous, humanities, and legal perspectives to campaign for the rights of nonhumans, humans, and the ecosystems they depend on (Rodríguez‐Garavito, 2024).

The “difficult work of better conceptualizing coexistence” Bruskotter et al. (2025) refer to cannot come from conservation scientists and affiliated Western experts alone. It is best codeveloped with those living with wildlife, and this includes individuals and communities and the institutions they have chosen to govern them.

Subsuming all these efforts under the banner of “effective conservation” as Bruskotter et al. (2025) do risks privileging wildlife over locals and delegating ultimate authority in this sphere to conservation scientists. This suggests anxiety over ceding authority to those who are not conservation scientists and the outcomes of doing so for wildlife.

I worry about this myself. For years, I have campaigned against the illegal farming of Ndumo Game Reserve (Pooley, 2025), South Africa, a case in which top‐down government has so far been at best apathetic. I am calling for top‐down interventions, providing these are done correctly, and initiate mediated processes with representatives of local communities.

In contrast to experiences in South Africa and in Gujarat, India, I have noticed an apparent managerialism in discussions of coexistence emanating from the European Union and the United States (e.g., Linnell et al., 2025; Vance Martin et al., 2021). This emerges from contexts where governments are regarded (by most) as legitimate and representative and where agencies have the authority, will, and resources to intervene. In certain developing world contexts, none of this is the case, and coexistence is in the hands of traditional authorities and local communities. Spiritual dimensions and cultural traditions may be just as (if not more) significant as scientific expertise or government policies and legislation (Holmes et al., 2018; Nijhawan & Mihu, 2020).

Procedural and outcome justice are intrinsically linked; inclusive and transparent processes are necessary for equitable outcomes. Perhaps Bruskotter et al. and I are not far apart on this. The difference may be that they want conservation scientists to decide what coexistence is and then to engage with other stakeholders, or risk “pursuing a misidentified target.”

These worries may emerge from immersion in a particularly divisive and destructive social world view regarding human relationships with the natural world (worries I wrestle with, too) and a conservation context far different from human–wildlife coexistence scenarios in other parts of the world (which have their own problems). In other words, some of Bruskotter et al.’s concerns and proposals may fit their contexts well, while being unsuitable for universal application. In regions where Indigenous knowledge and local traditions of coexistence with wildlife persist, the conservation approach should be collaborations among scientists, other engaged scholars and practitioners, and Indigenous and local experts and authorities (e.g., Miller et al., 2025).

I reiterate my general call for a positive vision for human–wildlife interactions through coexistence. Specific visions would need to be codeveloped for operationalization in particular scenarios. My hope is that an ongoing collaborative search will identify broad principles of coexistence, rather than impose universal standards. Collaboratively formulated with both conservation practitioners and those living with wildlife, and integrating Indigenous perspectives, these principles will help everyone concerned with human‐wildlife interactions support and encourage coexistence so that communities of humans and wildlife can flourish together.
